# An innovative and integrated model for global outbreak response and research - a case study of the UK Public Health Rapid Support Team (UK-PHRST)

**DOI:** 10.1186/s12889-021-11433-0

**Published:** 2021-07-12

**Authors:** Philomena Raftery, Mazeda Hossain, Jennifer Palmer

**Affiliations:** 1 Department of Global Health & Development and Health in Humanitarian Crises Centre, London School of Hygiene and Tropical Medicine, Keppel Street, London, UK; 2 Centre for Women, Peace & Security, London School of Economics and Political Science, Houghton Street, London, UK

**Keywords:** Outbreak preparedness and response, Operational research, Global Health governance, Global Health security, Epidemics, Emergencies, Innovation, Partnerships

## Abstract

**Background:**

Despite considerable institutional experimentation at national and international levels in response to calls for global health security reform, there is little research on organisational models that address outbreak preparedness and response. Created in the aftermath of the 2013–16 West African Ebola epidemic, the United Kingdom’s Public Health Rapid Support Team (UK-PHRST) was designed to address critical gaps in outbreak response illuminated during the epidemic, while leveraging existing UK institutional strengths. The partnership between the government agency, Public Health England, and an academic consortium, led by the London School of Hygiene and Tropical Medicine, seeks to integrate outbreak response, operational research and capacity building. We explored the design, establishment and early experiences of the UK-PHRST as one of the first bodies of its kind globally, paying particular attention to governance decisions which enabled them to address their complex mission.

**Methods:**

We conducted a qualitative case study using 19 in-depth interviews with individuals knowledgeable about the team’s design and implementation, review of organisational documents, and observations of meetings to analyse the UK-PHRST’s creation, establishment and initial 2 years of operations.

**Results:**

According to key informants, adopting a triple mandate (response, research and capacity building) established the team as novel in the global epidemic response architecture. Key governance decisions recognised as vital to the model included: structuring the team as a government-academic collaboration which leveraged long-term and complementary UK investments in public health and the higher education sector; adopting a more complex, dual reporting and funding structure to maintain an ethos of institutional balance between lead organisations; supporting a multidisciplinary team of experts to respond early in outbreaks for optimal impact; prioritising and funding epidemic research to influence response policy and practice; and ensuring the team’s activities reinforced the existing global health architecture.

**Conclusion:**

The UK-PHRST aims to enhance global outbreak response using an innovative and integrated model that capitalises on institutional strengths of the partnership. Insights suggest that despite adding complexity, integrating operational research through the government-academic collaboration contributed significant advantages. This promising model could be adopted and adapted by countries seeking to build similar outbreak response and research capacities.

**Supplementary Information:**

The online version contains supplementary material available at 10.1186/s12889-021-11433-0.

## Background

The Coronavirus-19 (COVID-19) pandemic is a stark reminder of how vulnerable the world is to pandemics and underscores the importance of investing in outbreak preparedness and response capacity. In recent years, a series of infectious disease threats have led to declarations of Public Health Emergencies of International Concern (PHEIC), and prompted reflection on how global health structures can best be designed to respond to epidemics [1]. The 2002–04 Severe Acute Respiratory Syndrome (SARS) pandemic, for example, prompted adoption of the 2005 International Health Regulations (IHR 2005) which state that all member states should maintain capacities to prevent, detect and respond to public health emergencies [2]. Prior to 2012, however, few countries were fully compliant [3, 4]. The scale of the West African Ebola epidemic in 2013–16, again highlighted the need to reform global health governance to address limitations of the global health community to both respond to, and conduct essential research in, complex outbreaks [5]. COVID-19, the largest of recent PHEICs, has uncovered several additional important issues. Theses include; tensions around capacity for domestic responses in high income countries versus the need for international assistance in low- and middle- income countries (LMICs); the importance and relevance of contextualised and localised response interventions; and the responsibilities of global bodies to ensure equitable access to the outputs of research and innovation, such as diagnostics, vaccines and treatments, among others [6, 7].

Despite considerable institutional experimentation and reform at the national and international levels to respond to calls for global health governance reform [1, 8–12], there is little research on organizational models that address epidemic preparedness and response. This paper contributes empirical material on the design, evolution and implementation of one such organization, the United Kingdom’s Public Health Rapid Support Team (UK-PHRST). Created in the aftermath of the response to the 2013–16 West African Ebola epidemic, the UK-PHRST was designed to address critical gaps in global outbreak response illuminated during the UK’s experience of responding to the Ebola epidemic in Sierra Leone, and to leverage cross-sectorial institutional strengths present in the UK.

### The global health response to the 2013–16 West African Ebola epidemic

The West African Ebola outbreak prompted an immense domestic and international response. While underlying political, social, and cultural factors within these countries enabled disease spread, weak health systems, poor domestic outbreak preparedness and a delayed international response were also responsible [1, 5, 10]. The World Health Organization (WHO) was criticised for its failure to acknowledge the potential severity of the outbreak and to coordinate an effective early international response [13]. Moreover, many of the world’s wealthiest nations were charged with ignoring the crisis until it directly threatened their own countries [13].

International deployment of technical staff was largely coordinated through the Global Outbreak Alert and Response Network (GOARN), a global network comprised of over 260 technical institutions and coordinated by an operational support team based at the WHO Headquarters, Geneva [14]. An estimated 2,500 international personnel were deployed from 40 organisations and 58 foreign governmental and non-governmental emergency medical teams from China, Cuba, the Africa Union, the United States, the UK and elsewhere [13, 15]. They worked alongside national medical and public health staff, and foreign and national military personnel, within Ministry of Health coordinated Incident Management Systems and treatment centres in the affected countries [3, 4].

Among foreign government donors, support for responses in the three most-affected countries was divided along historic colonial lines, enabling donors to build on existing partnerships and foreign relations infrastructure, with the UK taking the lead in Sierra Leone, the United States in Liberia, and France in Guinea [16]. In Sierra Leone, the UK’s leading role elicited coordination and contributions across UK government departments, including the Department for International Development (DFID) [now the Foreign, Commonwealth & Development Office (FCDO)], Public Health England (PHE), and the British military, as well as universities, and the private and civil society sectors, many of whom had long-standing ties to the country [15, 17].

The UK’s public health response in Sierra Leone was coordinated by the governmental agency PHE [15]. To staff the response on the ground, PHE seconded experts in public health, epidemiology, virology, clinical medicine and social sciences from government agencies and universities [15, 18]. Key UK organisations who deployed staff included the UK government’s Emergency Medical Team (UK-EMT), which typically responded to humanitarian crises; UK-Med, which supported deployment of National Health Service (NHS) clinical staff from the UK International Emergency Trauma and Medical Registers; Oxford University; King’s College London (KCL); and the London School of Hygiene & Tropical Medicine (LSHTM). Deployments tended to be channelled through organisations with an existing presence on the ground, such as the KCL Sierra Leone Partnership, and the UK sections of Save the Children and Médecins Sans Frontiers (MSF) and other non-governmental organisations (NGOs) [15, 19]. This enabled the UK response to build on existing infrastructure while facilitating service provision and training of national staff in surveillance, diagnosis and clinical management of Ebola cases [15, 19]. PHE also established laboratory capacity in Sierra Leone [20] and British military personnel contributed to building and staffing six Ebola treatment centres, including one dedicated to treating healthcare professionals, in addition to supporting overall coordination, logistics and training of health care workers [15, 16].

During the Ebola epidemic, UK institutions were also highly engaged in both operational research and research and development (R&D) of epidemic tools and technologies. Operational research findings drawing on mathematical modelling, epidemiological and anthropological research, for example, have been recognised as providing evidence that was pivotal for decision-makers to guide Ebola response policies, planning and intervention design in real-time [21–23]. UK partners also ran clinical trials with West African researchers, humanitarian agencies and the private sector to develop diagnostics, treatments and vaccines [15, 24]. While novel ways of conducting clinical research were devised, the UK, similar to most countries involved in the response, conceded they were unprepared to conduct clinical trials when the epidemic began [25, 26]. By the time most trials were implemented, numbers of positive cases had significantly reduced and clinical endpoints could not be met [15]. Examples include the PHE Defence Science and Technology Laboratory’s rapid diagnostic test for Ebola, which was developed, manufactured and trialled in Sierra Leone, but never operationalised [25, 26] and the US National Institute for Health trial of the immunotherapy ZMapp, which was stopped before reaching a definitive result [27]. This collective failure was seen as a missed opportunity to develop medical tools and technologies, that cost lives, compromised future responses and emphasised the need for advance preparation for future outbreaks [25, 26, 28].

### Redesigning the global health architecture post-Ebola

At a global level, widespread criticism of WHO’s role of the Ebola outbreak in a number of high-profile reports, influenced subsequent reforms of the global epidemic response architecture [1, 8–12]. Consensus recommendations included consolidating and strengthening WHO emergency and outbreak response activities, strengthening global disease surveillance and implementation of IHR core capacities, strengthening national health systems, and enhancing epidemic research and development [1, 10]. Actions taken since then have included the launch of the Global Health Security Agenda (GHSA) in 2014, which outlines specific actions that countries can take to meet IHR requirements [29] and development of a WHO Joint External Evaluation (JEE) tool in 2016 to facilitate collaborative assessment of countries’ IHR capacities [28, 30]. In 2016, the WHO Health Emergencies Programme (WHE) was created to enhance coordination and operational response capacity during health crises [31–33]. In parallel, the WHO Global Health Emergency Workforce, was established to facilitate rapid deployment of national and non-governmental EMTs to emergencies [33]. At regional level, the Africa Centers for Disease Control (Africa CDC) was established as a specialized public health institution of the African Union to strengthen the capacity of members states for infectious disease preparedness and response [34].

Encouraging investment has been made in research too. This includes development of the WHO Research and Development Blueprint, a global strategy and preparedness plan for rapid activation of research and development during epidemics, particularly for diagnostics, vaccines and treatments [28, 35], creation of a taskforce for operational research during outbreaks by GOARN and several public–private partnerships to foster research and development on infectious diseases [28, 36]. In addition, a variety of university-led knowledge hubs have been established to feed academic insight into epidemic decision-making [37].

In this study, we explore the creation of the UK-PHRST in the post-Ebola UK and global health policy context and examine early experiences of how the team implemented its mission as one of the first bodies of its kind. We pay particular attention to the governance decisions the UK-PHRST made which enabled them to address the complex mission. This study contributes qualitative empirical observations on the design, evolution and implementation of an integrated and innovative model for outbreak preparedness and response, which could inform other countries or organisations interested in developing such teams.

## Methods

### Timeline

The research took place in 2017–18, during Years 2 and 3 of the five-year UK-PHRST programme of work. When data collection began in September 2017, the UK-PHRST was transitioning from a protracted interim stage to the permanent phase of the project.

### Sampling

This study employed an embedded research approach using in-depth interviews, review of key documents and observation of meetings to gather data. Purposive sampling [38] was used to recruit participants knowledgeable on the research objectives. Nineteen in-depth semi structured interviews were conducted with individuals involved in the conceptualisation and establishment of the partnership [3], senior management team (SMT) [3], core management and core deployable team (CDT) involved in both the interim and permanent phase [5], representatives of key external stakeholders – Department of Health (DH), National Institute for Health Research (NIHR), DFID and GOARN [4] as well as members of the academic steering committee (ASC) and academic consortium [4].

### Data collection and analysis

In-depth interviews followed semi-structured interview guides (see Additional File 1: Qualitative Interview Guides) and were digitally recorded before transcription and analysis. An iterative process was applied whereby topic guides were edited following each interview to include questions on emerging themes in subsequent interviews. Key documents were reviewed and staff meetings and an induction day were attended to gain a deeper understanding of organisational objectives and policies. Framework analysis [39] was used to analyse data using NVivo 11 [40]. Despite the limited numbers of interviews conducted [19] saturation was reached on several key themes, which we outline below.

### Ethics

The London School of Hygiene and Tropical Medicine Observational / Interventions Research Ethics Committee reviewed and approved this research in September 2017, review reference No:14329 /RR/8906. All participants were informed of the study aims and objectives using a participant information sheet and all signed consent forms, including their chosen level of confidentiality.

## Results

### Designing the UK-PHRST within the UK policy context

The concept for the UK-PHRST can be traced to the second half of 2014, when institutions across the UK were ramping up activities in Sierra Leone in response to WHO’s August declaration that the Ebola epidemic had become a PHEIC. This was complemented by substantial political and scientific activity within the UK, including activation of the independent Scientific Advisory Group for Emergencies (SAGE) to channel scientific advice to the government emergency response committee [25, 26] and several informal meetings between Government departments and the university, civil society and private sectors. The idea for a rapid response team that could also conduct research emerged during discussions between LSHTM academics and staff of PHE and DH. The concept was championed by the UK’s Chief Medical Officer (CMO) and other advocates within DH, PHE, and the Prime Minister’s Office. The CMO proposed that a “rapid response force” like the UK-PHRST could strengthen PHE’s international public health functions [25, 26] and the team featured as a key deliverable of the PHE global health strategy delivery plan in January 2015 [41]. With the Ebola epidemic largely under control by mid-2015, the UK’s leadership and collective efforts were commended, and government began to reflect and address lessons learned [25]. Alongside other major commitments for infectious diseases research funding, including the substantial Ross fund, the UK-PHRST was formally announced at the G7 Summit in June 2015 by the UK’s Prime Minister [42, 43].

At the time, the UK-PHRST gained high-level appeal for a number of politically expedient reasons. Firstly, it married global health concerns with threats to domestic security. The overall integrated mission of the UK-PHRST, which was envisioned as the route to achieving the long-term outcomes and impact, was certainly outwardly focused on LMIC’s and humanitarian settings, to

“*prevent outbreaks from becoming public health emergencies, reduce morbidity and mortality*, *and ultimately make the world safer from outbreaks of infectious diseases”* [44] (p.6).

Nevertheless, early team strategy documents emphasised the health security benefits to the UK:

*“In addition to the benefits to stakeholders overseas, the UK-PHRST will help protect the UK population through the development of greater capacity to prevent, detect and respond to health threats internationally that might directly or indirectly affect the UK, resulting in potential health, economic or social harm.”* [44] (p.3).

The UK National Health Service (NHS) was also framed as better protected with a dedicated team of experts available to deploy to these epidemics, avoiding the complex and costly processes involved in ‘backfilling’ specialists from their day jobs [44]. Moreover, these experts would be funded by overseas aid money, meaning that the UK-PHRST would not be competing with the NHS for budget. While one DH representative acknowledged that any health project with an overseas focus was vulnerable to public criticism because:

“the Daily Mail and the Daily Mirror [UK newspapers] are obsessed with the aid budget”

they also reasoned that spending on international outbreak responses was more easily justified, saying:

“everyone can see that they [the team] go, they do. ‘Yay, the UK's got someone going off to deal with plague!’ ... that's an easier story to tell. UK expertise going out to help ... [for] the most paternalistic [reasons], that fits with their model of what the UK does”.

Secondly, research and innovation are important economies in the UK which are also embedded in the ways the Government work [44]. Giving the UK-PHRST a mandate to do research, therefore, made sense to stakeholders as it fitted with UK Government ideology of evidence-based interventions, while also addressing critical gaps experienced during the Ebola epidemic [25]. Having previously set up the NIHR, the UK’s CMO advocated for a strong academic component in this model, which laid the groundwork for the novel government-academic partnership.

*“It's because the CMO wanted an academic focus [...] it wasn't just about the doing, it was also about the research to improve the doing and then building on the capacity building stuff. The CMO wanted it to be rigorously academic. So, they [PHE] had to have an academic partner.”* (SMT member involved in conceptualisation).

Thirdly, the UK-PHRST’s concept furthermore contributed to a UK government strategy to pursue global impact through diffusing ODA spending across more domestic government departments and the higher education sector. Whilst being mindful that ODA funding exists to bring benefits to LMICs and helps grow international collaborations on a principle of equity, it also contributes to positioning UK institutions as leaders in the response to global challenges [45]. Many informants believed that the UK experiences in the Sierra Leone response should be harnessed in a project like the UK-PHRST, as described by a DH representative.

*“Because of the Ebola stuff, because we were so heavily involved in it, we have a global reputation for this sort of skill set. And leadership. . . we have a reputation. There is an expectation upon us. . . there is an assumption that the UK will engage”*

With the UK-PHRST organised to be ODA-funded, increased experience, technical capacity, and leadership skills of UK personnel was seen as a key selling point by authors of UK-PHRST strategy documents, which would also maximise synergy and effectiveness of other UK aid investments, including support to WHO [44].

For UK-PHRST team members and individuals closely involved in its set-up, however, the most important rationale for the team was its novel contribution to the global epidemic response architecture. With few governments in the Global North developing rapid response teams with specialist skills for epidemics (as opposed to wider skills for humanitarian emergencies, including natural disasters) and none explicitly incorporating a research component, they commonly described the UK model as “innovative”, “pioneering” and “pathfinding”. Team members furthermore hoped their model would catch-on in this expanding field, as described by this SMT member:

*“I think there’s a sort of initiative to have more of these teams set up and running [...]. I think we’re at the forefront of that wave, so I think we’re pathfinders to some extent. I hope that others will be [...] looking to us as an example and learning from what we’ve done.”*

### Implementing the UK’s integrated model for outbreak response

**Fig. 1:**
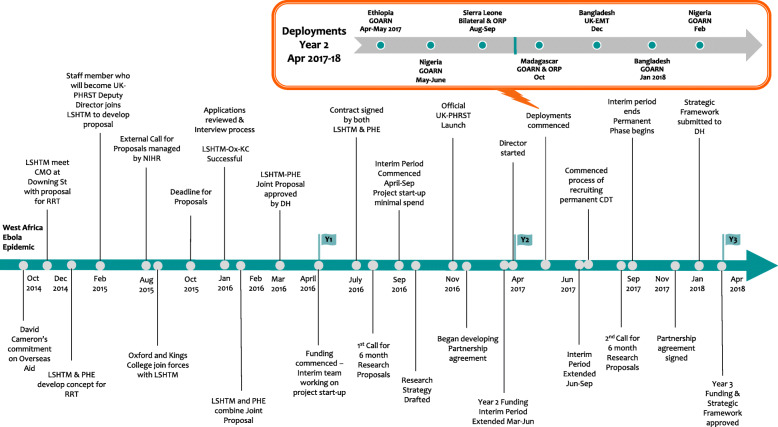
Timeline of events in the conceptualisation and establishment of the UK-PHRST. CMO = Chief Medical Officer, GOARN = Global outbreak alert and response network, PHE = Public Health England, LSHTM = London School of Hygiene and Tropical Medicine, UK-PHRST = UK Public Health Rapid Support Team, LSHTM-OX-KC = Academic consortium, ORP = Operational Research project. The deployments bubble represents the seven deployments which took place in year 2 from April 2017 to Feb 2018. Each deployment shows country of deployment, request type (GOARN, Bilateral or UK-EMT), whether an operational research project (ORP) took place during the response and the dates of the deployment. The initial three deployments took place during the interim phase of the project

The UK-PHRST team was considered a key component of UK cross-governmental global health security efforts, and was also expected to bridge key academic partners across the UK, as well as serve as an integral partner to the international community, especially WHO and GOARN, and other developing Rapid Response Teams [44]. Despite high-level enthusiasm to develop a national response team that combined response and research, however, the bureaucratic processes involved to get the UK-PHRST off the ground meant the team took 1.5 years to establish, and 3 years to fully operationalise. Meanwhile several more epidemics were detected globally, including the Zika epidemic, which was declared a PHEIC in February 2016. To document the journey from conceptualisation through to operationalisation of the team, we compiled a timeline of events presented in Fig. 1.

Although prior to Ebola, PHE had focussed on controlling outbreaks in the UK and acknowledged that it was not as well equipped to respond internationally to emergencies, they were the de facto government agency [25, 26]. LSHTM academics, who had been key members of the SAGE committee advising on government policy in Sierra Leone and had been involved in early discussions with Government on establishing a rapid response team, were obvious contenders. Building on their shared experiences in West Africa, Oxford University and KCL joined forces with LSHTM to form an academic consortium, contributing their expertise on clinical research and mental health towards a combined application. Following an external competitive process managed by NIHR, LSHTM and their partners were successful in January 2016, and subsequently merged their proposals with PHE. This disjointed process felt inefficient to observers and resulted in a year-long delay, as described by an ASC member involved in the UK-PHRST’s conceptualisation.

*“Well, PHE were obviously going to win it, right? So I don’t see any point of making PHE write an application separately [...]. I thought a more sensible thing to do would be to say to PHE . . . you should work with the shortlisted applicants to help them write a proposal that looks good [...]. It didn’t make any sense.”*

The long set-up period was compounded by further delays. While funding was released in April 2016, contracts were only signed in July and the official launch did not take place until November 2016. An interim period began in September 2016 with an administrative team and core deployable staff, whilst a full-time director and the permanent team were being recruited. Following a protracted recruitment process for the Director’s position, the post was occupied in April 2017 and work began on the strategic framework to guide the team’s long-term strategy. Recruitment of the core deployable team ran from June–September 2017 so the interim period was extended to cover this time. According to UK-PHRST personnel, the interim phase was characterised by substantial uncertainty and an unwillingness of the institutions to invest in team members as it was unclear if they would be hired as permanent staff.

*“During the interim phase it very much felt like a place holder [...] it felt like we had been put there because there was a rush to get the team in place and we needed to tick a box and tell Department of Health and the Government this rapid deployable team was there, ready to go.”* (CDT member)

The multi-disciplinary CDT consisted of three epidemiologists, two clinical researchers, a social scientist, two microbiologists, a data manager and analyst, an infection prevention and control expert and a logistician as well as the Director. A core management team supported the administrative aspects and the six-member senior management team consisted of the Director, Deputy Directors from PHE and LSHTM, the Microbiology Lead, a Senior Programme Manager at PHE, and a Programme Manager for LSHTM.

The governance and funding structures of the partnership split reporting responsibilities equally between the two key partner organisations. An annual lump sum was issued from the Treasury to DH for PHE, while for LSHTM, the team’s budget was managed as a research grant overseen by NIHR. Reporting on operational aspects of the grant followed the same DH-PHE and NIHR-LSHTM parallel arrangement. Ultimately, however, the UK-PHRST Director was accountable to the PHE Medical Director for delivery against the strategy and annual plans. PHE’s Medical Director then reported all UK-PHRST spending to DH, which was, in turn, accountable to the Treasury for the total amount, ensuring compliance to ODA funding rules. Because of the complicated governance structures and the fact that the programme was still in the early stage of operationalisation, reporting requirements felt cumbersome and complex for many members of the SMT with what was described as a three-step process.

*“It makes it more complicated because we have to report UK-PHRST activities to two different bodies which are interested in different things, and then those reports need to be stitched together so that there is a single report that goes back to DH [...] There’s certainly some duplication of work.”*

There was a perception by some PHE representatives that this split in funding added unnecessary complications and that it would have been more efficient if all funding had been channelled through PHE, who could then sub-contract LSHTM. Moreover, the inflexible nature of annual ODA funding was seen as a challenge both for implementing research and managing the unpredictable nature of outbreak response, as described by one individual involved in the conceptualisation.

*“It comes with all sorts of dire restrictions on what you can and can't do and how you report it, way, way beyond normal government funding. . . Because ODA funding is essentially David Cameron's [the UK Prime Minister’s at the time] promise to spend a certain proportion of GDP on international development, which became a legal requirement, therefore the ODA money has to be spent when it's given to you. And there's no flexibility by not spending it, you get heavily criticised by the Treasury if you don't spend it. 'Cause if you don't spend it, it means the UK doesn't meet its legal obligations under the act.”*

While the dual funding mechanism added complexity, it was nevertheless perceived as essential to maintaining organisational balance, as described by a member of the SMT.

*“I think if you ever look at sort of successful partnerships, particularly between different institutions in different places, if you think about international collaborations, if all the money goes through one party then they have all the power. And it’s not an equal relationship.”*

Despite the challenges and unanticipated delays encountered, the added value of the government-academic partnership was recognised by all involved as a significant collaborative advantage.

*“I think if this is simply something that is run out of a ministry and is entirely a civil-service driven thing, then I don’t think it would be nearly as exciting and viable.”* (SMT member)

### The role of the UK-PHRST in rapid outbreak response

Team members and observers believed the key strength of the UK-PHRST was its model of a standing, core team of experts who could deploy rapidly early in the outbreak to influence response activities and set up research early on in epidemics. This model also played to the team’s strengths, as described by one CDT member.

*“There are very few teams like us in the world, and so we need to use the fact that we are a small team of pretty experienced individuals. The best use of that skills is going very early. When you go late into an outbreak ... The difference you can make when you're part of a massive WHO operation is much less, and your ability to influence the course and the direction is much less.”*

Delays in establishing the permanent team meant that no deployments or research took place in year 1, and the year-1 budget went largely unspent. The first deployment took place in April 2017 and by April 2018, the team had deployed seven times, once bilaterally, once with the UK-Emergency Medical Team (UK-EMT) and five times through WHO’s GOARN (Fig. 1).

Deployment of the team to LMIC’s, was contingent upon receiving an official request for assistance from one of three sources: 1.bilateral requests directly from a national government, 2. indirect requests through GOARN or WHO regional or country offices, and 3. domestic requests to participate in UK humanitarian responses abroad, such as those led by the UK Emergency Medical Team (UK-EMT). With the UK-PHRST often described as a UK government ‘asset’, however, decisions surrounding the team’s deployment to LMICs involved sometimes complex political choreography, as acknowledged by one SMT member:

*“We can’t go anywhere unless we’re invited to go. Even if we are sitting on the sidelines and we’re seeing that an outbreak’s occurring, we think it’s important, and we could help there, we can’t go unless we’re invited.”* (SMT member).

Decisions surrounding the team’s deployments were ultimately subject to a cross-government decision protocol and ministerial approval, which included an assessment of the UK’s relationship with the requesting government, and was subject to ODA-eligibility. Informal communications through DH or UK-PHRST channels were often the initial alarm which would activate and inform a risk assessment of the public health case for intervention, which the UK-PHRST director would present to government ministers once a request was formally made. This included resolving potentially important political issues ‘behind’ multi-lateral requests. For example, before the team deployed to Ethiopia in 2017, according to a DH representative, Ministers considered the deployment in relation to the UK’s competition in concurrent elections for the Director General of the WHO and how that could affect their international relationships.

*“Tedros [the Ethiopian candidate] was competing with David Nabarro, who was our candidate [...] We didn't want to be seen to be going in [...] So, we always make sure there's a formal invite [...] so we know it is the government, not just WHO, who's invited us.”*

Deployments were then expected to happen within 48 h of a positive Ministerial decision, which team members saw as a significant advantage of the UK-PHRST over other global outbreak response mechanisms, particularly GOARN. Moreover, as described by a DH representative, the short-term nature of deployments (typically 4–6 weeks long) was viewed as critical to the teams added value, while more long-term response mechanisms, such as through GOARN, were being activated.

*“It's the business of having a standing team who can drop everything and go, because the one thing you can't do through GOARN, it's a process which requires you to put names forward, everyone thinks about it. They've got to backfill the posts. So, technically, they (UK-PHRST) can drop everything at 48-hour’s notice and off they go [...]So, that gives them enormous flexibility”*

The influence of the UK-PHRST abroad was also perceived to be partly dependent on the mode through which the team was deployed. Whereas bilateral deployments were experienced by team members as less bureaucratic and more efficient, key to fulfilling the mandate of rapid response, and research projects could be set up more easily, deployments via WHO-GOARN facilitated their contribution within the broader global health context. Members of the team felt that deploying to protracted outbreaks, such as to the Ethiopian cholera outbreak in April 2017, was not the best use of their capacities, while their contribution during acute emergencies such as the mudslides in Sierra Leone in August 2017 [46] and plague outbreak in Madagascar later that year, were considerably more meaningful. Despite the preference of some team members for bilateral deployments, a member of the SMT reiterated the importance of integrating with existing coordination mechanisms to avoid contributing to response fragmentation, an important lesson from the West Africa Ebola outbreak [5, 10, 12]. Moreover, team members were not branded as UK-PHRST when deployed through GOARN which was highlighted as potential tension.

*“There’s a delicate balance [...] we want the UK-PHRST to fit into that global architecture, and not be a part of a process causing confusion [...] versus being seen to be a UK asset, and the UK can fly the flag and show that they’re doing something globally.” (SMT member)*

### Conducting operational research in outbreaks

The UK-PHRST envisaged operational research as their most effective route to improving outbreak responses [44]. Whereas research on outbreaks is often deprioritised, delayed or separated from the early response for fear that it could detract public health resources, the explicit research component in the mandate meant that the UK-PHRST could prioritise setting up operational research alongside outbreak response in the early stages of an emergency. One individual involved in conceptualising the UK-PHRST described the importance of this for decision-making throughout an epidemic, based on his observations during Ebola:

*“I think it's important that we do research. But it's unbelievable the low level of evidence that goes into some of the decision making around these sort of emergency settings. [...] Ebola is a brilliant example of that. We treated nearly 30,000 cases of Ebola and if you look at the death rate it improved over the course of the outbreak from about 70, 80% in the outset to about 50% towards the end... we don't know why we improved the outcome. Because we didn't collect enough data [...] I personally think that's scandalous [...] we're the only one of the [Rapid response teams] that have been set up that does have a research component attached to it, an explicit research component.”*

The UK-PHRST research portfolio was divided into three distinct components: research during outbreaks, research in the immediate wake of outbreaks, and a long-term research agenda to be conducted outside of outbreaks. The research strategy was loosely organised according to five main streams covering epidemiology and population sciences, patient-centred research, microbiology and laboratory sciences, social sciences and community engagement, and mental health and wellbeing, which capitalised on existing expertise and research interests of scientists at the universities, many of whom were involved in the Ebola response [44, 47]. The research strategy also identified priority pathogens, such as Ebola, Lassa Fever, Marburg and Vector borne diseases, which aligned with the research strategies of other groups, including WHO (R&D Blueprint), Coalition for Epidemic Preparedness Initiative (CEPI) and the UK Vaccine Initiative (UKVI). To ensure rigour, the team set-up an academic steering committee (ASC) made up of experts from participating UK-PHRST institutions to evaluate and select research proposals in alignment with the research strategy and organisational objectives. The team envisaged the research streams being linked, with data from the operational research being used to inform real-time decision making.

By the end of year 2, two rounds of standalone, short-term (6–12 month) research projects had received funding and two operational research projects had been set up during deployments to outbreaks. Study investigators came from the core team as well as academic staff from the collaborating institutions and research studies spanned disciplines and included multiple diseases. (Details of UK-PHRST publications available at: https://www.lshtm.ac.uk/research/centres-projects-groups/uk-phrst#publications). Some examples of short-term research projects conducted in the early stages included; developing a rapid review methodology for clinical research to identify research gaps quickly in an outbreak; examining the quality of clinical characterization in refugee camps in Greece as a component of outbreak detection and characterization; and an audit of the quality of the Ebola data that was captured in West Africa. The mental health component included a project which explored the train the trainers model in cognitive behaviour therapy for anxiety and depression amongst Ebola treatment centre health workers.

While many observers praised the team for getting research studies off the ground quickly, since primary responsibility for research lay with the universities and operational response with PHE, several respondents also felt that this separation was hindering greater integration of research within responses.

*“I think it’s great that the RST is probably the only and the first initiative that really tries to give an equal weight to research and the public health response, which is fantastic. At the same time, the responsibilities have been split [between lead institutions], which still keeps them a bit divided [...] I think we should be aspiring towards greater integration of the research with the public health response.”* (Member of ASC)

In theory, all core team members were expected to contribute to delivering on the triple mandate (response, research and capacity building) and to play each role of responder, researcher and trainer throughout the short deployments and in ‘peacetime’ (when not on emergency deployments). In reality, however, managing implementation of the triple mandate by individual team members was believed to be challenging. It was accepted that outbreak response would take precedence over the operational research programme, so research continuity plans would be required, however they were not yet drafted at the time of the research. These plans were expected to define responsibilities of the wider faculty at the host academic institutions for maintaining research programmes whilst CDT members were responding to outbreaks or conducting urgent research. While approvals for setting up research projects in the midst of an outbreak worked well, respondents did highlight the challenges of conducting research during short-lived outbreaks.

*“In terms of applying for the funding, we didn't have to apply for the Madagascar funding. We asked the RST if there were funds available to do an emergency research project and there were, because there are reserve funds for that. Getting the okay for that was actually very quick, very easy, very simple. That's what you need in an outbreak. You don't need a lengthy, drawn-out process where you have to formally apply for funding.[...] A major issue was recruiting enough patients. Even though we managed to get off the ground in two weeks, the outbreak was winding down by the time we started.” (CDT member)*

Given that ODA funding needed to be spent on an annual basis, research projects needed to be modest and respondents reported particular difficulties navigating approval processes including ODA justification, setting up studies and maintaining staff contracts to fit within these 6–12-month timeframes. While respondents referred to the development of a more long-term, multidisciplinary research strategy with rolling multi-year workplans, as of Year 2 (2018), this had not yet been elaborated and was highlighted as a gap.

*“I was thinking that we would have something that would say over the next four years, these are the five big questions we want to answer, but maybe I'm being overly optimistic, maybe it is better to do it opportunistically.”* (Member of ASC)

Research budgets were quite modest and always understood to be insufficient to fund large-scale studies with approximately £500,000 available per year. Annual amounts needed to be flexible, depending on the demand on the overall budget of the team’s response to outbreaks, which was unpredictable. Around 15% of the annual operational research budget was earmarked for emergency research in the context of an outbreak, with the remaining 85% expected to be spent on the long-term research agenda. The scheme was expected to fund pilot studies which could be continued or scaled up using external funding, with the short-term projects acting as the ‘spark’ needed to attract larger investments in epidemic research and build collaborations. However, at the time of data collection, some academic observers were sceptical that this strategy would be effective.

*“I think the short-term projects are, by definition, they’re not sustainable [...] one of the ideas was that the research activities would be sustained by getting supplementary funding from other sources. That’s easier to do if you’ve got demonstrated achievements in a sizeable project that’s complex enough, that there’s multiple secondary research activities.”* (Member of academic consortium)

Acquiring external funding to support the research portfolio was perceived to require additional team investment in building partnerships in host countries and sourcing funding. Generating and applying evidence through operational research was described by one SMT member as a route to catalysing policy change:

*“I’m really all about evidence-based ways of doing things. Then, it gives you a strong platform for policy change.”*

However, respondents felt that the work required to ensure that evidence could be disseminated effectively and translated into policy and practice had been underestimated in the initial years of the team’s work, particularly the investment required to work on this with local actors and implementers on the ground.

*“Any research has to have a strong local engagement, a capacity development component, adequate recognition, very assiduous care about intellectual property, data ownership and sharing, sample ownership and sharing.”* (Member of ASC)

### Capacity building for outbreak response

The third component of the UK-PHRST mission was training and capacity-building in LMICs to enable countries to respond effectively without international support. A key goal stated in the strategy was *“to ultimately eliminate the need for its existence”*, however at the time of the research this was the least evolved components of the mandate with a lack of clarity on the priorities and strategies for implementation. The following quote of an SMT member demonstrated the aspirational and long-term vision of the UK-PHRST to develop sustainable human resource capacities and build national response mechanisms in LMICs.

*“We want to have it [a situation] 20 years from now where people would say, "Well, why the hell would you have the UK respond to an outbreak in Africa? They have that capacity." We want to help build that capacity.”*

Capacity building was conceptualised as a continuous process required throughout an outbreak response where international staff work side-by-side with national responders to develop their skills and capacities. Providing technical advice and on-the-job mentorship to national responders was a large part of the work of international responders when on deployment. Nevertheless, it was recognised by team members that there was a limit to the capacity building they could do in the short-term deployments and that building more long-term systems was the role of WHO, which has a long-term presence at country level in many LMIC’s.

*“We worked on the emergency surveillance system in Sierra Leone. My previous team were there for two weeks, I was there for five weeks. What you see is a mass of holes in the normal surveillance system. I can be there for two months or five months or ten months and I'm not going to fix that. It takes years and that's what WHO is for.” (CDT member)*

At the time of data collection, a key strategy of the UK-PHRST was to establish regional hubs for research and capacity-building internationally, the first of which was planned in Sierra Leone and would build on the connections developed by PHE and the universities during the Ebola outbreak. The overseas sites were expected to provide a platform for capacity building and enable the development of regional hubs for teaching and research [44]. A number of scholarships for short courses and Masters in Public Health were offered to individuals in Sierra Leone and other LMICs through the UK-PHRST academic institutions and members of the team occasionally lectured at universities in Sierra Leone. Team members were also expected to spend large parts of their time operating out of these hubs. In practice, however, members of the deployable team felt that being based in LMIC’s would create challenges for other aspects of their mandate and would compromise team cohesion [48].

*“I don't want to be based permanently somewhere else. Partly because part of the strength is having the school around you[...] I think if we're going to have a team you actually have to have your team based somewhere. If you base your nine people, three here and three there and three there, then they're not a team anymore, you can't really function as a team.” (CDT member)*

Since this research was undertaken, this strategy had been abandoned and the concept of regional hubs is no longer being pursued.

The UK-PHRST strategy also involved an element of UK domestic capacity-building to amplify their expertise through training a cadre of ‘reservists’ intended to expand the capability within the UK to rapidly scale-up responses to larger outbreaks and epidemics. Reservists and members of the UK field epidemiology training programme (FETP) were expected to be trained to UK-PHRST standards and protocols and available to deploy when necessary while retaining their routine “day jobs”. The aim was to deploy reservists at least once every 2 years to maintain their skills and to build their experience, as well as to ensure their continued interest and engagement. In addition, the UK-PHRST sought to play a leading role with WHO and GOARN in developing standardised trainings for international response teams.

## Discussion

The UK-PHRST was established in 2016 in the wake of the Ebola epidemic, to address one of the global health community’s most critical challenges, integrating operational research and capacity-building with rapid outbreak response. We describe the design, creation and evolution of the UK-PHRST within the UK and global health policy context since its establishment post-Ebola, and report qualitative reflections on the challenges, successes and lessons learned as they implemented the complex triple mandate. While our findings are specific to the UK context and the ways in which the UK conceptualises their role in the global health architecture, this case study could offer insights on the process of designing and establishing such organisations in other countries, which we outline below and discuss in relation to the current COVID-19 pandemic.

### Designing and implementing the UK’s integrated and innovative model

Conceptualised by public health actors in the UK who were intimately involved in addressing the challenges presented by the West African Ebola epidemic, the UK-PHRST combined expertise from the government and academic sectors to address the need for rapid outbreak response, supported by an integrated research approach. The unique triple mandate assigned to them established the UK team as innovative in the global epidemic response architecture but involved many domestic, international and institutional governance challenges which they faced in the inception period. To our knowledge, in this evolving and expanding sector, the UK-PHRST remains the only team globally to systematically prioritise and integrate operational research alongside rapid response to outbreaks, reflecting a long tradition of evidence-based policy-making in the UK. This organisational model provides an example that could be adapted by other governments seeking to establish similar mechanisms. Demonstrating their successes and achievements, alongside descriptions of how they solved structural challenges, may even drive establishment of such teams at a local level in LMICs, particularly in Africa where more than 150 public health emergencies occur each year [49, 50].

The UK-PHRST had to reflect, course-correct and evolve in their early days. The process of establishing the partnership, for example, was beset by unanticipated bureaucratic delays reflecting the challenges of establishing a government-academic partnership, while also choosing ways of working which capitalised on each institution’s strengths and fitted the UK Government’s approach to overseas assistance. The initial vision of the UK-PHRST emphasised equal power dynamics between lead organisations and a commitment to operating as one cohesive team. Despite this, both funding and governance structures were separated across the two main organisations. Reporting requirements were perceived by many team members to be cumbersome and complex with annual financial disbursements described as inflexible and restrictive especially for research, causing potential tensions between the two lead organisations. Some even suggested that a subcontract between PHE and LSHTM would have been more efficient, given the power dynamics in reality, which leveraged PHE’s existing fit within government structures. Regardless, it was clear that the rationale behind this separation was to maintain the ethos of institutional balance between organisations, as defined during conceptualisation of the partnership.

In exploring the genesis and evolution of the UK-PHRST, it was evident that the team’s mandate to conduct research was important in helping garner high-level support for its creation. The policy idea of ‘evidence’, and that investments in public programmes should contribute to generating evidence, was appealing to decision-makers in the UK context and to UK institutions. The restrictive format of how ODA funding needs to be disbursed and accounted for in the UK, however, structured implementation in a way that sometimes constrained the team’s collaborative working and long-term planning. The story of the UK-PHRST is therefore one about how the appeal of evidence influences political and financial decision-making, but also of how political and financial structures in turn influence the evidence that can be produced.

While integrating the three objectives was key to the collaborative advantage and innovative role of the UK-PHRST, in reality, implementing the triple mandate has been challenging, as core team members straddle the roles of responder, researcher and trainer [48]. The research and capacity-building components required greater facilitation through multidisciplinary collaborations, partnering with institutions in the host countries and a high level of contingency planning to ensure research continued when team members were on deployment. Moreover, capacity building priorities and strategies were not well elaborated in the initial stages, reflecting the need to prioritise deployments and research activities [51] and the decision to abandon the strategy of regional hubs may have been influenced by the demand which the triple mandate placed on team members.

### The role of the UK-PHRST in rapid outbreak response

Experience shows that a rapid response to infectious disease outbreaks can stop transmission, preventing minor outbreaks from becoming major epidemics. However, an important limiting factor for outbreak control in LMICs is having a skilled workforce with the required expertise in public health, epidemiology, clinical management, laboratory skills, social sciences and other relevant disciplines [28] and maintaining a functional roster of trained staff available to deploy [3]. The UK-PHRST considered their primary strength to be their model as a standing, small team of multi-disciplinary experts able to deploy early in the response preventing outbreaks from spiralling out of control. Remaining within this remit was considered important for the team to demonstrate their added value within the broader global health architecture, providing short-term, immediate support, while GOARN coordinates the longer-term deployments, which often involves recruitment of experts with existing employment commitments. Emphasising the importance of this, a recent review of national public health RRT’s management highlighted several challenges in the development and maintenance of an RRT roster to ensure deployable surge staff were selected, available to deploy, trained and had the relevant competencies to be effective in the field [3].

Team members’ perceptions of their impact also depended on the mode of their deployment. Bilateral deployments were preferred by some team members as they seemed to enable more effective implementation of the triple mandate by allowing more autonomy to set up research projects and to build relationships with academic groups on the ground, as well as closer working relationships with national response staff. However, the team’s leadership ultimately prioritised reinforcing and positively influencing the existing global architecture for outbreak preparedness and response by working closely with WHO, GOARN and stakeholders and governments in affected countries. Understandably, this created tension between visibility and recognition as the UK-PHRST versus alignment with the WHO-led response, which was also highlighted by a recent mid-term evaluation of the UK-PHRST [51]. In future, such relationships with existing global and national institutions will likely be instrumental in amplifying the impact of the UK’s investment and the ability of the UK-PHRST to translate their expertise and the evidence they generate into policy and practice.

Epidemic preparedness efforts to date have largely focussed on the model of high-income countries supporting LMICs where most infectious disease outbreaks happen, and this is the model which the UK-PHRST also adopted. However, the COVID-19 pandemic is challenging this model as response mechanisms of even the wealthiest countries, including the UK, have been overwhelmed. The ability and willingness of high-income countries to support LMICs in the COVID-19 pandemic has been severely curtailed by overburdened health systems in high-income countries and enforcement of international travel restrictions [7]. In the early stages of the pandemic, for example, following requests from WHO, Africa CDC, and national governments, the UK-PHRST deployed team members to the Philippines, Nepal, Ethiopia, Bangladesh and Nigeria but subsequent travel restrictions forced the UK Government to recall team members. Moreover, despite the efforts of the UK-PHRST to identify and train a large pool of reservists to serve in epidemic response, the need for these reservists to work in their day jobs largely prevented their deployment. During the West African Ebola epidemic too, although global and UK institutions made unprecedented efforts to mobilise and second their staff, policies such as flight bans and quarantines threatened to undermine response efforts [13]. While UK-PHRST members continued to provide remote COVID-19 support in Africa and Asia, the effectiveness of remote support at country level is understandably limited and building local capacity for outbreak response in LMICs remains the most effective and sustainable model.

### The importance of conducting operational research in outbreaks

A fundamental lesson from the West Africa Ebola outbreak was the critical need for scientific evidence that can only be generated by conducting research during the response [52]. The COVID-19 pandemic has vastly increased momentum and improved coordination in the field of R&D compared to previous epidemics [7, 53, 54]. Through the Access to COVID-19 Tools Accelerator (ACT-Accelerator), WHO have strongly advocated for global coordination and collaboration to harness research investments into global solutions while ensuring equity of access [55]. Moreover, while research on epidemic response has historically tended to take a narrow biomedical approach dominated by epidemiologists, clinicians and microbiologists, the COVID-19 pandemic has shone a light on the complexity of epidemics and the importance of evidence-informed policy [6]. The range of policies being implemented across different countries reflect different needs for, and interpretation of, available evidence in different political, social and cultural contexts, highlighting the importance of political and social science insights and contextually-adapted solutions [6, 56]. Maintaining a multi-disciplinary emphasis in epidemic research plans of teams like the UK-PHRST, which incorporates insights from anthropology, political science, economics and other social science disciplines, would be important to inform real-time policy decisions [1, 10, 12]. By example, the UK-PHRST are well positioned to draw upon the broad expertise of the academic partners involved in the partnership to elaborate and implement a research strategy that can truly inform response efforts.

Our findings on the UK-PHRST demonstrate that establishing collaborative research partnerships in LMICs and co-defining research priorities with partners on the ground, can not only ensure the relevance of research findings, but can also bridge the gaps between evidence generation, policy and practice, a key objective of operational research [57]. Ensuring access to populations, clinical specimens and public health data requires partnering with governments and local actors in host countries who may not prioritise research among other competing health system challenges. For those establishing teams like the UK-PHRST, we recommend proactively engaging host governments, universities and public health actors in priority countries to promote research collaborations and facilitate dissemination and translation of research findings [52, 57]. For the UK-PHRST, the support of the three universities’ (LSHTM, Oxford, KCL’s) overseas research sites meant that when setting up projects they could draw upon pre-existing infrastructures and partnerships within the well-established overseas networks of collaborators, a significant opportunity and advantage of marrying the organisations. For the UK-PHRST, ODA funding restrictions had to be navigated and placed constraints on the partners, impacting effective implementation of the research strategy. For other RRT’s, building flexibility in the funding mechanism is an important lesson, and defining a strategy that is aligned with global priorities [35] is likely to encourage external funding commitments and build sustainability of the research portfolio.

### The UK-PHRST's contribution to building global health security

In West Africa, it was estimated that the cost of the Ebola crisis was three times higher than it would have been to invest in preventative public health systems in the affected countries [5, 58, 59]. General consensus that the only fail-safe strategy for limiting the impact of epidemics, is building resilient health security systems in every country, has been heavily reinforced by the COVID-19 pandemic [1, 28, 60, 61]. The UK-PHRST supports this aspiration by building outbreak response capacity in LMICs and using operational research evidence to inform context-specific policies and practice. Major investments and significant progress has been made since Ebola on strengthening global health security, through the completion of JEE assessments in over 100 countries and subsequent development of national action plans for health security by more than 60 countries [61]. Since 2015, WHO’s Health Emergencies Programme has developed and validated open access, standardized RRT training material and has trained over 2,000 public health professionals [62]. In reality, however, building strong health security systems in LMIC’s will require addressing broader governance issues, including implementation of the IHR core capacities, bolstering leadership and management capabilities, and eliciting commitment of governments to fund national health security initiatives [33]. Under their present model, UK-PHRST members recognised that it is not feasible for the team to address all of these challenges, which requires continuous in-country presence, contextual understanding and strong relationships with government counterparts, ultimately the role of WHO. Therefore, it is important that they work closely with WHO country offices and other UK programmes, including the PHE IHR strengthening projects, as well as other partners on the ground to ensure their contribution is coordinated within national action plans for health security.

Given their limited capacity coupled with the complex mandate of the UK-PHRST it would be prudent to focus capacity building efforts on their niche areas of expertise, namely: i) developing and implementing a standardised model for rapid response team training in LMICs, ii) developing and delivering pre-deployment training programmes for international responders with GOARN, iii) capacity-building for operational research, including training academics and students in LMICs to develop and implement research protocols, set up clinical trials and conduct observational studies, ensuring research can be conducted and led by local investigators, and iv) offering international educational opportunities in the UK to strengthen public health workforces in LMICs. Together these would ensure that they can optimally contribute to an epidemic preparedness and response workforce, building on demonstrated UK institutional strengths, while avoiding duplication with WHO and other partners.

### Study limitations

Data for this study was collected early in the establishment of the UK-PHRST, primarily during a period of transition from the interim to a more permanent phase, limiting the number of relevant staff available for interview and introducing potential bias based on accessibility of interviewees. Only 19 people were interviewed out of a much larger samples size, including the full UK-PHRST team of 16 individuals, the ASC and multiple external stakeholders. Nevertheless, these respondents were purposively sampled to get a broad range of perceptions including members of the team from all four institutions, the ASC and key external stakeholders heavily involved in the design and/or implementation. Some members of the team and several external stakeholders were not included due to the limited scope and timeframe of the study. Information was nevertheless triangulated and validated to reduce bias and address gaps.

## Conclusion

The importance of investing in global outbreak preparedness and response capacity has never been more apparent, as highlighted by the COVID-19 pandemic [6]. The 2013–16 Ebola outbreak drove establishment of the UK-PHRST partnership at a time when political will for global health reform was strong. The UK recognised that rapid response by the international community to outbreaks was insufficient and needed to be combined with expansion of research capabilities and strengthened response capacity in LMICs [49]. Many unanticipated challenges emerged throughout the early stages of operations which the UK-PHRST had to navigate by developing, revising, abandoning or upholding key governance decisions. While some were specific to the UK context, others may be more generalisable and included: 1) structuring the team as a government-academic collaboration which built on long-term UK investments in public health and the higher education sector and leveraged the complementary knowledge, relationships and infrastructure available within partner institutions; 2) maintaining the ethos of institutional balance between government and academic organisations which required adopting a more complex, dual reporting and funding structure; 3) supporting a standing team available to rapidly respond to outbreaks early, on request, preventing outbreaks from spiralling out of control; 4) prioritising and funding the research component both during outbreak response and in “peacetime” to generate evidence that could influence policy and practices of existing and future epidemics; and 5) ensuring epidemic response and capacity-building activities reinforced the existing WHO-led global health security architecture.

The UK-PHRST aims to enhance global outbreak preparedness and response using an innovative and integrated model that capitalises on UK institutional strengths. Our research suggests that despite adding complexity, integrating operational research through the government-academic collaboration contributed significant advantages and remains a promising model for responding to the ongoing global threat of epidemics. This integrated model could be adopted and adapted by countries seeking to build similar capacities.

## Supplementary Information

**Additional file 1.** Qualitative Interview Guides used for semi-structured interviews.

## Data Availability

The datasets analysed during the current study are available from the corresponding author on reasonable request.

## Abbreviations

ACT-Accelerator: Access to COVID-19 Tools Accelerator
Africa CDC: Africa Centres for Disease Control
ASC: Academic Steering Committee
CDT: Core deployable team
CMO: Chief Medical Officer
COVID-19: Coronavirus-19
DFID: Department for International Development
DH: Department of Health
EMT: Emergency medical team
FCDO: Foreign, Commonwealth & Development Office
FETP: Field epidemiologists training programme
GHSA: Global Health Security Agenda
GOARN: Global outbreak alert and response network
IHR: International health regulations
JEE: Joint External Evaluation
KCL: Kings College London
LMICs: Low-middle income countries
LSHTM: London School of Hygiene and Tropical Medicine
NGO: Non-Governmental Organisation
NHS: National Health Service
NIHR: National Institute for Health Research
ODA: Overseas Development Assistance
PHE: Public Health England
PHEIC: Public Health Emergency of International Concern
R&D: Research and development
SAGE: Scientific Advisory Group for Emergencies
SMT: Senior Management Team
UK: United Kingdom
UK-PHRST: UK Public Health Rapid Support Team
WHE: WHO’s Health Emergencies Programme
WHO: World Health Organization
