# Supplementary material for: An innovative and integrated model for global outbreak response and research - a case study of the UK Public Health Rapid Support Team (UK-PHRST)
Source: BMC Public Health. 2021 Jul 12;21:1378. doi: 10.1186/s12889-021-11433-0 (PMC8273030; doi:10.1186/s12889-021-11433-0)
Supplement: Supplementary file 1 — Additional file 1. Qualitative Interview Guides used for semi-structured interviews. [file 12889_2021_11433_MOESM1_ESM.pdf]

Additional File 1: Qualitative Interview Guides

Objective: To gather information on the conceptualisation, mission, governance, leadership, partnership management and outbreak response and research conducted to date of the UK-PHRST.

The interview topic guides are structured as follows:

1) Vision, Mission and Conceptualisation
2) Leadership, Management and Partnerships
3) UK-PHRST Outbreak Response Operations and Research
4) Externa Stakeholder Relationships

Topic Guide 1: Vision, Mission and Conceptualisation

Vision, Mission and Conceptualisation

1. Briefly describe your role within the context of the UK-PHRST?
2. What is the vision of the UK-PHRST and how was this conceptualised?
3. Describe the mission of the UK-PHRST?
4. What was the driving force behind the conceptualisation of the programme?
5. How long did the process from conceptualisation to operationalisation take? Briefly describe the process and your role.
6. What were the milestones and challenges experienced along the way?
7. Was the partnership envisioned from the beginning, how was this envisioned to work? How does that differ from the reality?
8. How does the partnership enable or act as a barrier to the UK-RST fulfilling its mandate?

Governance, Funding and Partnerships

9. Briefly describe the organisational structure of the UK-PHRST and how it fits within the broader global health context?
10. How is the programme governed and how does it interact with other UK government departments? Dept of Health, DFID etc
11. How do you see the ethos of equal partnerships being maintained, governed and managed within the UK-PHRST?
12. How was the programme funded and what funding was received over what time period?

13. Briefly describe how funding is streamed through the programme.

14. What are the existing policies governing operations and influencing decisions around what outbreaks to respond to and what research projects are funded?

15. What do you see as the internal factors (e.g. political, policy, programs), which may impact the success of the UK-PHRST? ie. Strengths and Weaknesses

External environment and Key Stakeholder Relationships

16. What do you think are the external factors influencing the strategic direction of the programme?
17. Who are the key external stakeholders and how does the programme interact with the external stakeholders
    a. UK stakeholders and context
    b. International actors and context
    c. National governments and partners

18. How does the programme complement or integrate with the existing global health/emergency response mechanisms and organisations? . . . such as GOARN and DFID

19. What do think are each key stakeholder's INTEREST LEVEL and INFLUENCE/POWER LEVEL (low, medium or high) regarding the priorities of the UK-PHRST?

20. What do you see as the external factors (e.g. political, policy, programs), which may impact the success of the UK-PHRST? ie. opportunities and threats

Conclusion

21. Do you have anything else to add? Suggestions? Recommendations?

22. Are there other individuals or organizations you can suggest I speak with regarding the UK-PHRST?

Topic Guide 2: Leadership, Management and Partnerships

Mission, Strategy and Key priorities

1. Describe your role within the UK-PHRST?
2. What is the overall mission of the UK-PHRST programme?
3. What are the priorities of the programme related to
    a. Preparedness and response for emergencies
    b. Operational Research

c. Training and capacity building

4. Are you aware of the strategic direction or strategic plan for the UK-PHRST and were you involved in the strategic planning process? Please describe the process.

5. What are the policies that govern decisions around what emergencies are responded to and what research projects are funded?

6. How many and what emergencies have already been responded to and why? Explain the process and decision making.

7. How is funding decided and allocated to selected projects?
    a. Emergency operations
    b. Research projects

Management, Leadership and Internal Partnerships

8. Briefly describe the organisational structure of the UK-PHRST and how it fits within the broader context?

9. What are the accountability mechanism and structures for reporting on progress and financial management?

10. How are the partnerships governed and managed with in the UK-PHRST
    a. PHE and LSHTM, Oxford University, Kings College
    b. LSHTM and Oxford University and Kings College

11. How is accountability ensured within the partnership?
    a. Accountability
    b. Power sharing
    c. Conflict/compatibility
    d. Trust
    e. Expectations – compliance with expected roles

12. What communications and meetings exist for collaboration and communication between organisations involved in the partnership?

13. What do you see as the internal factors (e.g. political, policy, programs), which may impact the success of the UK-PHRST? ie. Strengths and Weaknesses

14. How would you describe the leadership style used within the organisation?

15. What is your understanding/opinion of the conceptualisation as a partnership rather than a separate entity.

16. How is the partnerships governed and managed with in the UK-PHRST between two entities with very different organisational cultures, principles, protocols etc.?

Value and Sustainability

17. What do you see as the added value of the UK-PHRST?

18. What do you think have been the successes/achievements to date?

19. What do you think have been the challenges to date?

20. Do you experience challenges of the funding mechanism – lack of flexibility, annual, short term projects? How does this compare with similar programmes?

21. How is sustainability being addresses

    a. In relation to the continuation of the programme

    b. In relation to the emergency response operations?

    c. In relation to the research and capacity building components of the programme?

External environment and Key Stakeholder Relationships

22. What do you see as the external factors (e.g. political, policy, programs), which may impact (barrier/enablers) the success of the UK-PHRST? ie. opportunities and threats

23. What do you think are the external factors influencing the strategic direction of the programme?

24. Who are the key external stakeholders and how does the programme interact with the external environment?

    a. UK stakeholders and context

    b. International actors and context

    c. National governments and partners

25. How does the programme complement or integrate with the existing global health/emergency response mechanisms and organisations such as GOARN and DFID?

26. What do think are each key stakeholder's INTEREST LEVEL and INFLUENCE/POWER LEVEL (low, medium or high) regarding the priorities of the UK-PHRST?

Conclusion

27. Do you have anything else to add? Suggestions? Recommendations?

28. Are there other individuals or organizations you can suggest I speak with regarding the UK-PHRST?

5) Topic Guide 3: UK-PHRST Outbreak Response Operations and Research

Individuals Deployed on Emergency Response Missions

1. Briefly describe your role within the UK-PHRST.

2. Have you been deployed as part of an emergency mission? Where and what was the emergency response mission

3. Briefly describe the process involved in the deployment, did you find it efficient and effective?

4. How do you think these processes could be streamlined or improved?

5. What do you see as the challenges of the deployment?

    a. Comment on timing and length of deployments.

6. What do you see as the successes of the mission?

7. What do you see as the added value of the UK-PHRST during outbreak response?

8. What training did you receive prior to deployment?

9. Did you feel adequately supported and trained to fulfil your mandate?

10. How is sustainability being addresses in relation to the emergency response operations?

11. Please explain the training and capacity building component of the programme?

    a. Please explain the concept of host centres?

<u>Those leading operational research projects</u>

12. Briefly describe your role within the UK-PHRST and the research project you are involved in?

13. How to you see your research aligns with the mission and strategic direction of the UK-PHRST?

14. Briefly describe the process involved in applying for funding for your research project through the UK-PHRST. Was it effective and efficient?

15. How do you think these processes could be streamlined or improved?

16. What do you see as the challenges for the project?

17. What do you see as the successes of the project?

18. What do you see as the added value of the UK-PHRST in the broader Global Health context?

19. How is sustainability being addresses in relation to the research and capacity building components of the programme?

<u>Conclusion</u>

20. Do you have anything else to add? Suggestions? Recommendations?

21. Are there other individuals or organizations you can suggest I speak with regarding the UK-PHRST?

Topic Guide 4: External Stakeholder Relationships

1. Briefly describe your role and your involvement with UK-PHRST.

2. What do you think are the external factors influencing the strategic direction of the programme (politics, policies, partners)?

3. Describe your organisations relationship with UK-PHRST?

4. Describe your organisations impression of and interaction with UK-PHRST.

5. How does the programme complement or integrate with the existing global health/emergency response architecture?
    a. UK stakeholders and context
    b. International actors and context
    c. National governments and partners

6. What is your organisations INTEREST LEVEL and INFLUENCE/POWER LEVEL (low, medium or high) regarding the priorities of the UK-PHRST?

7. What do you see as the external factors (e.g. political, policy, programs), which may impact the success of the UK-PHRST? ie. opportunities and threats

8. What do you see as the added value of the UK-PHRST?

9. What do you see as the internal factors (e.g. political, policy, programs), which may impact the success of the UK-PHRST? ie. Strengths and weaknesses

10. Do you believe sustainability being addresses in relation to the continuation of the programme?

Conclusion

11. Do you have anything else to add? Suggestions? Recommendations?

12. Are there other individuals or organizations you can suggest I speak with regarding the UK-RST
